# Soluble solids content prediction of pineapple based on visible-near infrared hyperspectral image

**DOI:** 10.3389/fpls.2025.1758676

**Published:** 2026-01-21

**Authors:** Yanli Yao, Junjun He, Zhangyun Gao, Zhuying Zhu, Shenghui Liu, Chuanling Li, Hui Feng, Xiumei Zhang

**Affiliations:** 1 Key Laboratory of Tropical Fruit Biology, Ministry of Agriculture (Zhanjiang), Key Laboratory of Hainan Province for Postharvest Physiology and Technology of Tropical Horticultural Products, South Subtropical Crops Research Institute, Chinese Academy of Tropical Agricultural Sciences, Zhanjiang, China; 2 National Key Laboratory of Crop Genetic Improvement, National Center of Plant Gene Research (Wuhan), Hubei Hongshan Laboratory, Huazhong Agricultural University, Wuhan, Hubei, China

**Keywords:** feature extraction, hyperspectral technology, pineapple, regression models, soluble solids content

## Abstract

Pineapple is widely favored by consumers for its rich proteins, vitamin C and other nutrients. Soluble solids content (SSC) has long been the core indicator for pineapple quality assessment, directly affecting its market acceptability and sales. To accurately detect pineapple SSC, this study used a hyperspectral imaging system to collect hyperspectral images in the 400–1700 nm range, with SSC measured by an Atago PAL-1 digital sugar meter as the reference. Five pretreatments (including multiple scattering correction (MSC), polynomial smoothing (SG) and mathematical transformations) were applied to raw spectral data, and three prediction models (partial least squares regression (PLSR), Lasso regression, ridge regression (RR)) were established. All models performed well: PLSR showed R²=0.9459 and RMSE = 0.5746, Lasso R²=0.8965 and RMSE = 1.0221, RR R²=0.8560 and RMSE = 1.2632. After screening characteristic bands via Successive Projections Algorithm (SPA) and re-modeling, the ddA-PLSR model was optimal (R²=0.9869, RMSE = 0.1250), with four key wavelengths (673-676nm, 711-715nm, 971-990nm, 1357-1367nm) extracted. This confirms hyperspectral imaging (HSI) enables efficient and accurate SSC detection in pineapples, with great application potential in pineapple quality identification.

## 1 Introduction

Pineapple (Ananas comosus) is one of the four major tropical fruits in the world. It is favored by consumers due to its unique flavor, rich nutrients such as sugar, dietary fiber, and vitamin C in the pulp, and its health care functions such as reducing blood pressure and promoting digestion (Ali et al., 2020; Abraham et al., 2023; Li et al., 2024). Pineapple is native to the high temperature and dry tropical areas of South America. At present, there are about 80 pineapple-producing countries in the world, among which Asian countries account for about 60% of the total output (Hossain, 2016). China has a long history of pineapple cultivation, with the planting area and total output accounting for about 7.4% and 8.3% of the global total, respectively (FAO, 2023). It is a highly efficient tropical cash crop and an important economic source for farmers in hot areas (Li et al., 2024).

The measurement of soluble solids (SSC) is a conventional parameter used to assess the quality of pineapples, which significantly influences their grading and sales (Rahim et al., 2014). Currently, pineapple grading methods primarily rely on visual assessment or destructive chemical techniques, resulting in unnecessary waste and low efficiency and accuracy (Semyalo et al., 2024). Therefore, the exploration of an accurate and rapid nondestructive detection method for determining pineapple SSC not only establishes a scientific foundation for assessing internal quality but also holds significant implications for advancing the pineapple industry.

Non-destructive testing is an emerging interdisciplinary application field that utilizes various physical methods, such as heat, sound, light, electricity, and magnetism, to obtain the quality, properties, and composition of the tested items without damaging them (Li et al., 2016, Li et al., 2019; Yu et al., 2022). Currently, the main methods of non-destructive testing for fruit quality include near-infrared spectroscopy, hyperspectral imaging, intelligent sensory biomimetics, acoustic characteristic, and electrical characteristic non-destructive testing technology (Guo et al., 2016; Munera et al., 2017; Aghilinategh et al., 2020; Yang et al., 2020b; Yu et al., 2022). Hyperspectral imaging technology is an optical-based imaging technique that can simultaneously acquire spectral information from multiple wavelength ranges to obtain detailed information about the target object (Lu et al., 2020). Due to its simplicity, continuous operation without interference, high accuracy and efficiency in image acquisition and analysis over a wide range of wavelengths, it has shown great potential in evaluating fruit quality (Lu et al., 2020; Wei et al., 2020; Zhang et al., 2020; Ma et al., 2022).

There have been many studies on using hyperspectral imaging technology to quickly and non-destructively detect the internal quality of fruits, including apples, pears, grapes, mangoes, cherries etc (Reddy et al., 2021; Tian et al., 2023; Wang et al., 2023);. Reddy et al. (2021) employed hyperspectral imaging and multivariate statistical methods to detect the soluble solids content in cherries. They established predictive models using Partial Least Squares Regression (PLSR) and Gaussian Process Regression (GPR). The test set results demonstrated that the GPR model achieved an R² of 0.88 and a Root Mean Square Error of Test (RMSET) of 0.43 for predicting cherry soluble solids content (Reddy et al., 2021). Wang et al. (2023) used hyperspectral imaging technology to detect fruit defects in cherries non-destructively, with an accuracy rate of 91.43% (Wang et al., 2023). Xu et al. (2023) used hyperspectral imaging technology to collect image and spectral information of grapes, and established partial least squares (PLS) and least squares support vector machine (LS-SVM) prediction models for total soluble solids (TSS) and titratable acidity (TA) based on the extracted feature spectra. The results showed that the SAE-LS-SVM deep learning model could achieve rapid and non-destructive detection of TSS and TA in grapes (Xu et al., 2023). Tian et al. (2023) used visible light and near-infrared (400–1000 nm) hyperspectral imaging technology to evaluate the quality of mangoes, and the results showed that the partial least squares (PLS) regression model established based on the CARS method and the selected feature variables had a coefficient of determination of up to 0.9001 on the prediction set, a root mean square error of 0.6162, and could effectively predict the soluble solids content (SSC) of mangoes (Tian et al., 2023).

Currently, there have been many studies on using hyperspectral technology to establish mathematical models for non-destructive prediction of the internal quality of apples, pears, grapes, etc., while there have been few reports on using hyperspectral technology to detect the quality of pineapples (Dong and Guo, 2015; Ardila et al., 2020; Xu et al., 2023; Yang et al., 2020a). This study uses 100 pineapple fruits as experimental samples and uses various regression methods to build prediction models to achieve non-destructive detection of the soluble solids content of pineapples, providing a scientific method for non-destructive detection.

## 2 Materials and methods

### 2.1 Pineapple samples

The experimental materials were collected from the Pineapple Experimental Base of the South Subtropical Crops Research Institute, Chinese Academy of Tropical Agricultural Sciences. A total of 5 mature fruit varieties for fresh consumption were collected, with the specific quantities as follows: 7 fruits of ‘Watermelon’, 17 fruits of ‘Josapine’, 23 fruits of ‘Tainung No.23’, 26 fruits of ‘Tainung No.16’, and 27 fruits of ‘Tainung No.21’. After being numbered and labeled, the pineapple samples were placed in the hyperspectral image acquisition laboratory 24 hours in advance for temperature equilibration. This step ensured that the internal temperature of each pineapple sample was consistent with the room temperature, thereby reducing the interference of temperature on both spectral data collection and physicochemical property determination.

### 2.2 Hyperspectral imaging system

**Figure 1:**
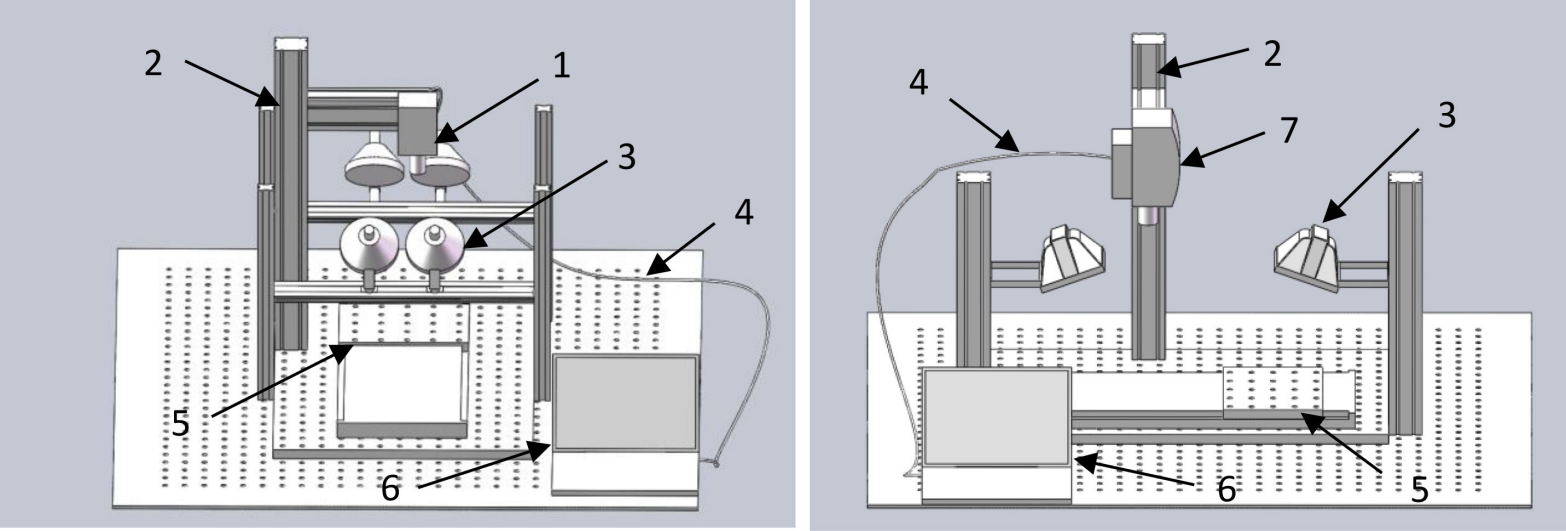
Hyperspectral imaging system:(1) Visible light camera (400-1000nm); (2) Aluminum alloy bracket; (3) Light source; (4) Optical fiber; (5) Conveyor; (7) a PC with image acquisition soft; (7) Near infrared camera (900-1700nm).

The hyperspectral imaging system comprises an imaging spectrometer (HyperspecTM VNIR, with EMCCD, Headwall Photonics, USA) featuring an EMCCD sensor with a pixel count of 1004*1002 and a pixel size of 8×8 μm^2^, capable of capturing data at 14 bits per pixel. Additionally, the system includes a halogen lamp (150W; Holac; Philips Lighting Co., Ltd., Shanghai), a custom conveyor (Wuhan Hongxingyang Technology Co., Ltd., China), and a computer (B365M TIGER GENERAL Ver1.0, HP, USA) (**Figure 1**). The visible light spectral range spans from 400 to 1000 nm with a resolution of 2–3 nm, while the near-infrared spectral range covers 900 to 1700 nm at a resolution of 4.65 nm.

### 2.3 Spectral acquisition and preprocessing

**Figure 2:**
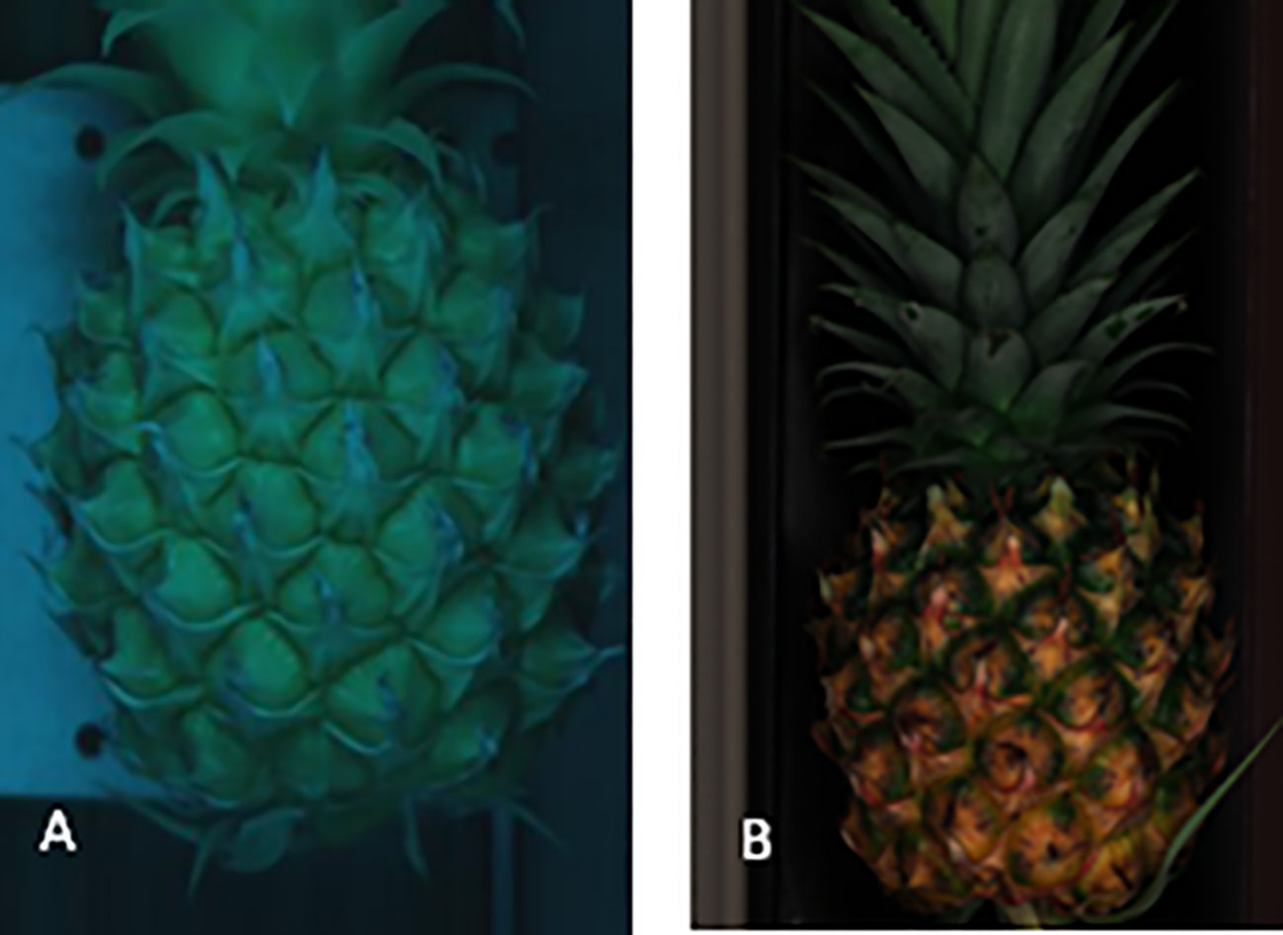
Visualization Images: **(A)** Scanning image in visible light; **(B)** Scanning image in near infrared.

**Figure 3:**
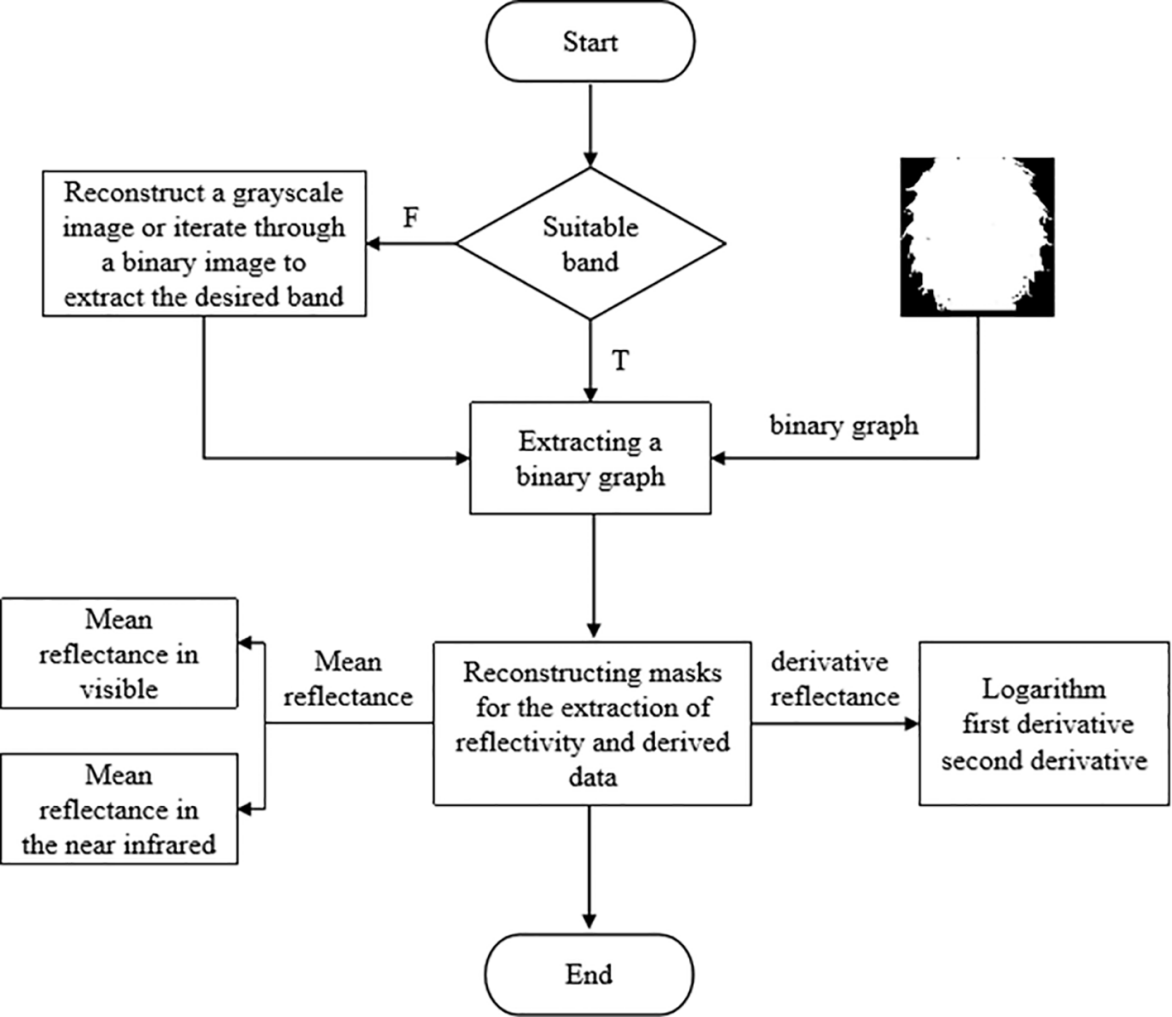
Data extraction flow chart.

The acquisition site of hyperspectral images in this study is the entire sample surface. The acquisition of hyperspectral image data is performed using the hyperspec software (Spectral Imaging Ltd., Finland). Prior to spectral data acquisition, adjustments should be made to the field of view, setting the exposure time to 30ms, determining the speed of the electrically controlled displacement platform to be within a range of 20mm/s, and positioning the lens at a distance of 40 cm from the transmission platform in order to ensure clear and undistorted images. During data acquisition, the linear array detector performs horizontal scanning in the vertical direction of the optical focal plane to capture image information for each pixel across various wavelengths within the scanning space. Simultaneously, as the electrically controlled displacement platform advances, it enables complete scanning of the entire plane by means of linear array detector (**Figure 2**), which is then transmitted via USB interface for storage on a computer. The spectrographic camera is sensitive to light and noise, so whiteboard and dark current correction are used. After the collection of the original spectral data is completed, a binary image reflecting the ROI area is produced through deep learning methods. The average reflectance of all the pixel points in the ROI area is extracted as the reflectance value of each band. The process for data extraction is illustrated in **Figure 3**.

Due to the presence of external interference information and noise in the spectral data collected through hyperspectral acquisition, such as electrical noise, artificial noise, and stray light, the accuracy of spectral prediction is affected by spectral baseline movement and drift. Therefore, effective preprocessing methods are selected to enhance the prediction accuracy of the model (Faber and Nicolaas, 1999). Multivariate scattering correction (MSC) can effectively reduce scattering effects in the spectral data obtained, thereby improving absorption information related to component concentration (Zhang et al., 2021). Savitzky-Golay (SG) filtering method enhances spectrum smoothness and reduces noise influence (Shi and Yu, 2017). The derivative algorithm (D^1st^) efficiently mitigates baseline drift and background interference in spectra. Consequently, this study employs three preprocessing methods (MSC, SG smoothing, and D^1st^) to process original spectra collected for constructing partial least squares regression (PLSR), Lasso regression, and ridge regression (RR) models respectively (Alcin et al., 2014). Furthermore, a comparison is made between these three pretreatment methods along with untreated spectral data to establish an optimal prediction model.

### 2.4 Soluble solids content measurement

The soluble solids content of pineapple was measured by Atago PAL-1 digital display sugar meter, which has a measuring range of 0-53% Brix, a resolution of 0.2% Brix and a temperature automatic compensation function. The specific method was to peel the pineapple, take the upper, middle and lower pulp of the fruit respectively, squeeze the juice to cover the mirror of the sugar meter, and directly read the value from the window. To ensure the accuracy of the measurement results, each sample was measured three times, and the average of the three measurements was taken as the final soluble solids content value for record. After each measurement, the mirror surface of the refractometer was thoroughly cleaned with distilled water to avoid cross-contamination and ensure the accuracy of the next measurement.

### 2.5 Sample division methods

For establishing a stable and widely applicable spectral prediction model, it is crucial to select an appropriate sample splitting method. After sample splitting, one part is used for model training, and the other part is used to evaluate model performance. In general, it should be ensured that the training set samples are evenly distributed, and the data range of the test set samples should be covered as comprehensively as possible. In this study, random splitting method is selected for modeling evaluation. This approach is suitable for various data sets and model types as it effectively mitigates overfitting issues associated with specific data splitting methods, thereby enhancing the model’s generalization ability (He and Ma, 2015).

### 2.6 Feature band screening method

When a hyperspectral camera is used to collect spectral information of each continuous band, each spectral information contains a large number of data points, which contain correct information reflecting the content of soluble solids, but also redundancy, collinearity and many noise information. If the full-band spectrum is used for modeling and regression prediction, the modeling efficiency will be reduced, and these overlapping and noise information will also affect the model accuracy. Therefore, in order to build a simpler, more robust and even more accurate model, this paper uses the Successive Projections Algorithm (SPA) (Li et al., 2016), combined with MSC, SG and D^1st^ three spectral preprocessing methods, to build a prediction model for pineapple fruit metabolites based on characteristic band spectrum.

### 2.7 Model establishment and evaluation

#### 2.7.1 Prediction models based on partial least squares regression, least absolute shrinkage and selection operator regression and ridge regression

##### 2.7.1.1 PLSR

PLSR (Baiano et al., 2012; Xie et al., 2018) is a multivariate statistical regression method often used to handle high-dimensional and multicollinear independent variable data. Its core idea is to extract a set of new latent variables based on the correlation between independent variables (X) and dependent variables (Y), so that these latent variables can not only explain the main variance of the independent variables but also maximize the correlation with the dependent variables. Compared with traditional multiple linear regression, PLSR can effectively overcome the problems caused by multicollinearity and show better robustness when the sample size is relatively small or the number of variables is large.

The key parameter of the model is the number of latent variables (i.e., the number of principal components, denoted as n_components), whose value will directly affect the model’s fitting ability and generalization performance. To determine the optimal number of latent variables, this study set the range of latent variables (n_min = 2, n_max = 30) to find the fundamental relationship between two matrices (X and Y), i.e. a hidden variable approach to model the covariance structure in these two spaces, where the independent variable X is the selected band of the hyperspectral index and the dependent variable Y is the value of the pineapple metabolite SSC. PLSR will attempt to find the multidimensional direction in X space to explain the maximum variance multidimensional direction in Y space.

##### 2.7.1.2 LASSO regression

LASSO regression in machine learning is a regression method that introduces an L1 regularization term on the basis of traditional linear regression. This method achieves constraint and sparsification of feature coefficients by adding a penalty term for the absolute value of coefficients to the loss function, thereby simultaneously realizing feature selection and parameter estimation during the model fitting process (Kabir et al., 2024). Among them, alpha is the regularization parameter, which is used to control the intensity of the penalty term. When alpha is large, some regression coefficients in the model will be compressed to zero, thereby achieving variable screening. When alpha is relatively small, the model approaches ordinary least squares regression (OLS). Therefore, the selection of alpha has a crucial impact on the model’s performance. In this study, in order to determine the optimal regularization parameter alpha, sets the search scope at the alpha (10 ^ 5, 1.0), and USES the logarithmic interval traverse generate 50 candidate values. For each alpha, a LASSO model is constructed separately to find the optimal alpha parameters.

##### 2.7.1.3 RR

Ridge regression method is a biased estimation method for analyzing multicollinear data, which was first proposed by Hoerl and Kennard (Hoerl and Kennard, 1970). Its essence is a regression process that gives up the unbiasedness and partial accuracy of least squares method and seeks for a slightly worse effect but more consistent with the actual situation, which can simplify the model and improve the robustness of the model. Ridge regression method punishes the complexity of the model by adding an L2 regularization term, reducing the risk of overfitting (Fan et al., 2023). The regularization parameter alpha controls the strength of the regularization term, and its value range is usually from 0 to positive infinity. A larger alpha value means a stronger penalty, which prompts the model parameters to tend towards 0. In this study, appropriate alpha values are selected for the different numbers of independent variables in the full band and characteristic bands to achieve a balance between model complexity and prediction performance, and to improve the stability and generalization ability of the model.

#### 2.7.2 Model evaluation

The model’s performance is typically assessed using corresponding indicators. In this study, the prediction model is quantitatively evaluated based on the Coefficient of Determination (R2), Root Mean Square Error of Prediction (RMSEP), and Mean Absolute Percentage Error (MAPE) for both measured and predicted values. A higher R2 value indicates a stronger correlation between the two datasets (Formula 1). A lower RMSEP value suggests greater stability in the model (Formula 2). A smaller MAPE value reflects higher prediction accuracy of the model (Formula 3). The calculations are presented in Formulas (1), (2), and (3).

(1) $$R^{2}=\left[1-\frac{\sum_{i=1}^{n}{\left(y_{i}-{\hat{y}}_{i}\right)}^{2}}{\sum_{i=1}^{n}{\left(y_{i}-\bar{y}\right)}^{2}}\right]\times 100\%$$

(2) $$MAPE=\frac{1}{n}\sum_{i=1}^{n}\frac{\left|y_{i}-{\hat{y}}_{i}\right|}{y_{i}}$$

(3) $$RMSEP=\sqrt{\frac{1}{n}\sum_{i=1}^{n}{\left({\hat{y}}_{i}-y_{i}\right)}^{2}}$$

Where n is the sample size; y_i_ is the measured value of the i-th sample; $\hat{y}$_i_ is the predicted value of the i-th sample; $\bar{y}$ is the average value of the measured sample.

## 3 Results

### 3.1 Statistical analysis of SSC in pineapple samples

This study adopted the method of fixed random seeds for dataset division to ensure the reproducibility of the results. As shown in **Table 1**, the descriptive statistics of SSC (including range, mean and standard deviation) of the training set and the test set are basically consistent. This result verifies the balance of data division, indicating that the training set fully covers the overall data characteristics, while the test set can serve as a fair benchmark for evaluating the generalization ability of the model.

**Table 1:** Statistics of soluble solids content in pineapple samples.

Set	Seed	Number	Maximum value/%	Average value/%	Mean%	Standard deviation
Calibration	42	90	23.85	10.37	17.3333	3.1770
Prediction	42	10	22.87	11.82	17.9380	3.4545

### 3.2 Vis-NIR spectroscopy analysis

**Figure 4:**
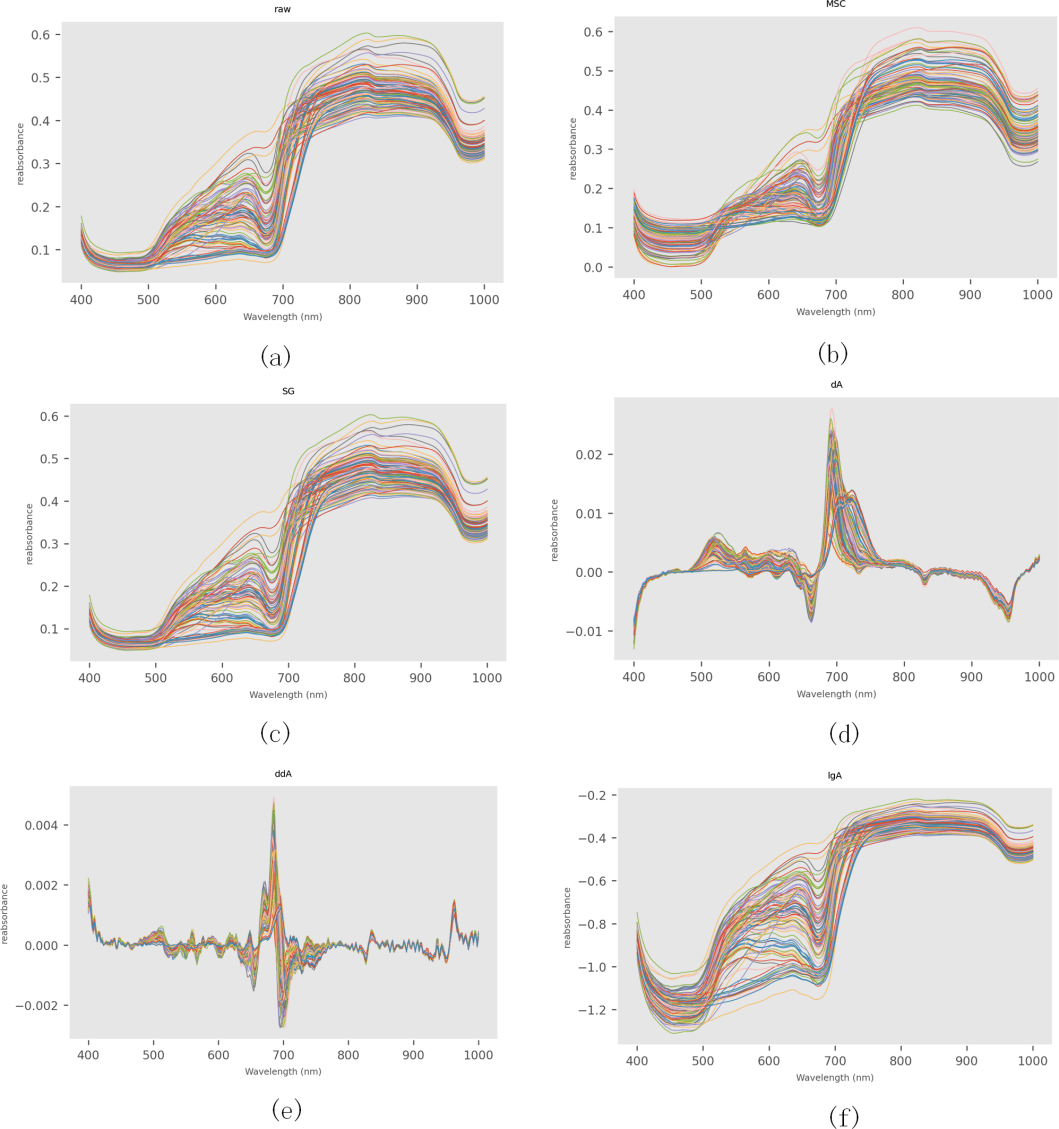
Visible-light wavelength SSC metabolite preprocessing spectrogram. **(a)** Original visible-light wavelength spectral curve diagram; **(b)** Visible-light wavelength spectral curve diagram after MSC processing; **(c)** Visible-light wavelength spectral curve diagram after SG processing; **(d)** Visible-light wavelength spectral curve diagram after taking the first derivative; **(e)** Visible-light wavelength spectral curve diagram after taking the second derivative; **(f)** Logarithmic visible-light wavelength spectral curve diagram.

**Figure 5:**
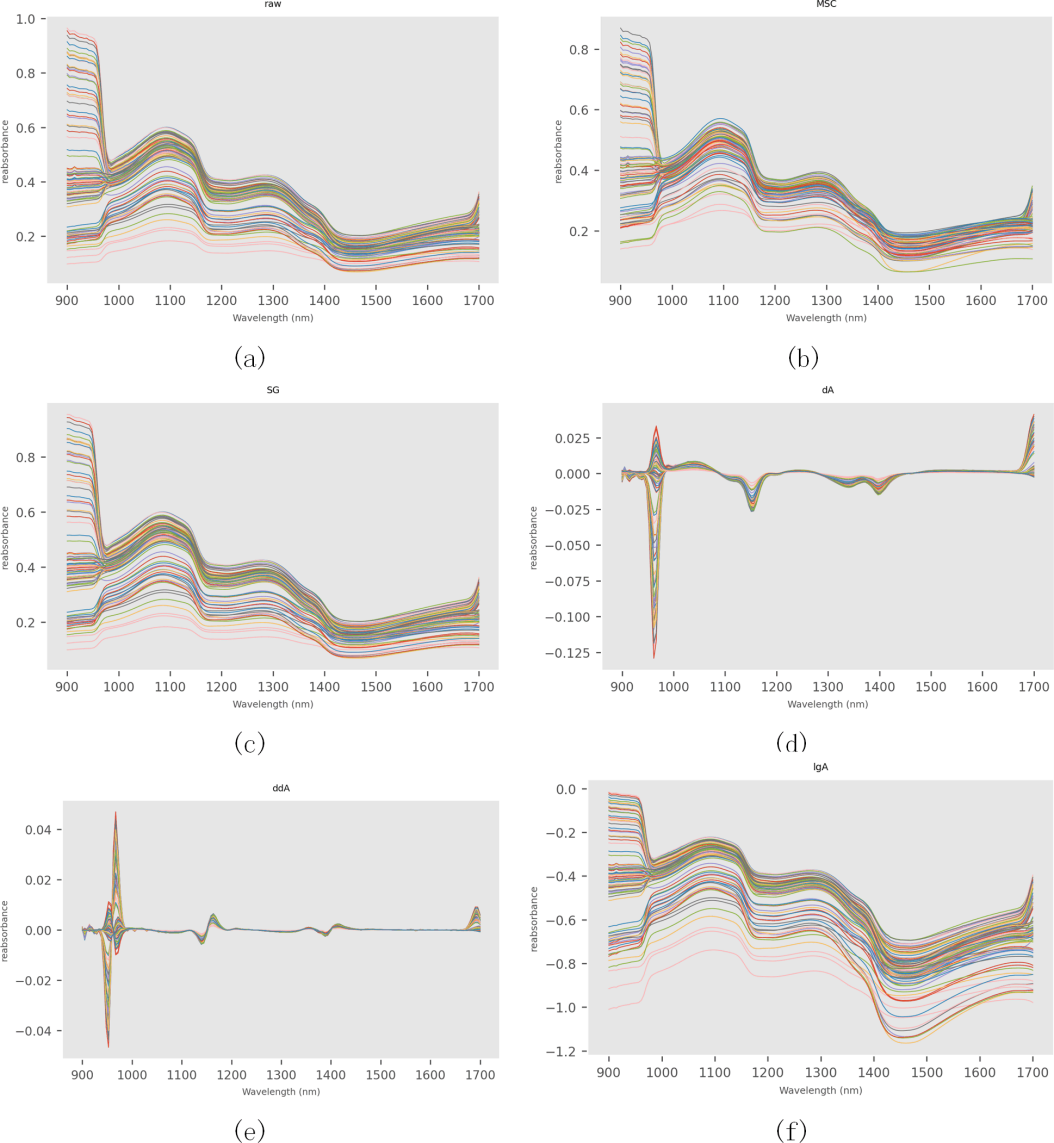
Near-infrared spectral pretreatment of SSC metabolites. **(a)** Original near-infrared spectral curve of SSC metabolites; **(b)** Near-infrared spectral curve of SSC metabolites after MSC pretreatment; **(c)** Near-infrared spectral curve of SSC metabolites after SG pretreatment; **(d)** Near-infrared spectral curve after taking the first derivative; **(e)** Near-infrared spectral curve after taking the second derivative; **(f)** Logarithmic near-infrared spectral curve of SSC metabolites.

The spectral reflectance can be obtained from the calibration hyperspectral image, and the relationship between the spectral reflectance and wavelength can be obtained. **Figures 4a**, **5a** are the original visible/near-infrared spectral curves of 100 pineapple samples. It can be seen from the figure that there is basically no difference in the trend of spectral curves of each sample, and there are no obvious abnormal samples. In the range of 400-500nm, the spectral curve is smooth and the reflectance value changes little. After 690nm, the reflectance of the samples increased rapidly, with peaks of 810, 910, 1100 and 1300nm and troughs of 690, 980, 1180 and 1420nm. There was an obvious absorption peak at 680nm, which was mainly caused by chlorophyll absorption spectrum on the surface of pineapple fruit (Choo, 2019; Huang et al., 2022; Wang et al., 2015; Yan et al., 2021). The absorption peak at 980nm is caused by the strong absorption of water molecules (Shinzawa et al., 2011; Goke, 2018; Yuan et al., 2024), which reflects the water content information of pineapple fruit in this band. Due to the influence of light wave scattering and migration, it is not easy to obtain spectral reflection feature points and their corresponding wavelengths. In order to eliminate their influence, The methods of multiplicative scatter correction(MSC), SG smoothing and derivation and logarithm transformation (first derivative (dA), second derivative (ddA) and logarithm (lgA)) were applied respectively to preprocess the spectral data and obtain the corrected spectra (**Figures 4b-f**, **5b–f**). .

### 3.3 Selection of characteristic spectral bands

**Figure 6:**
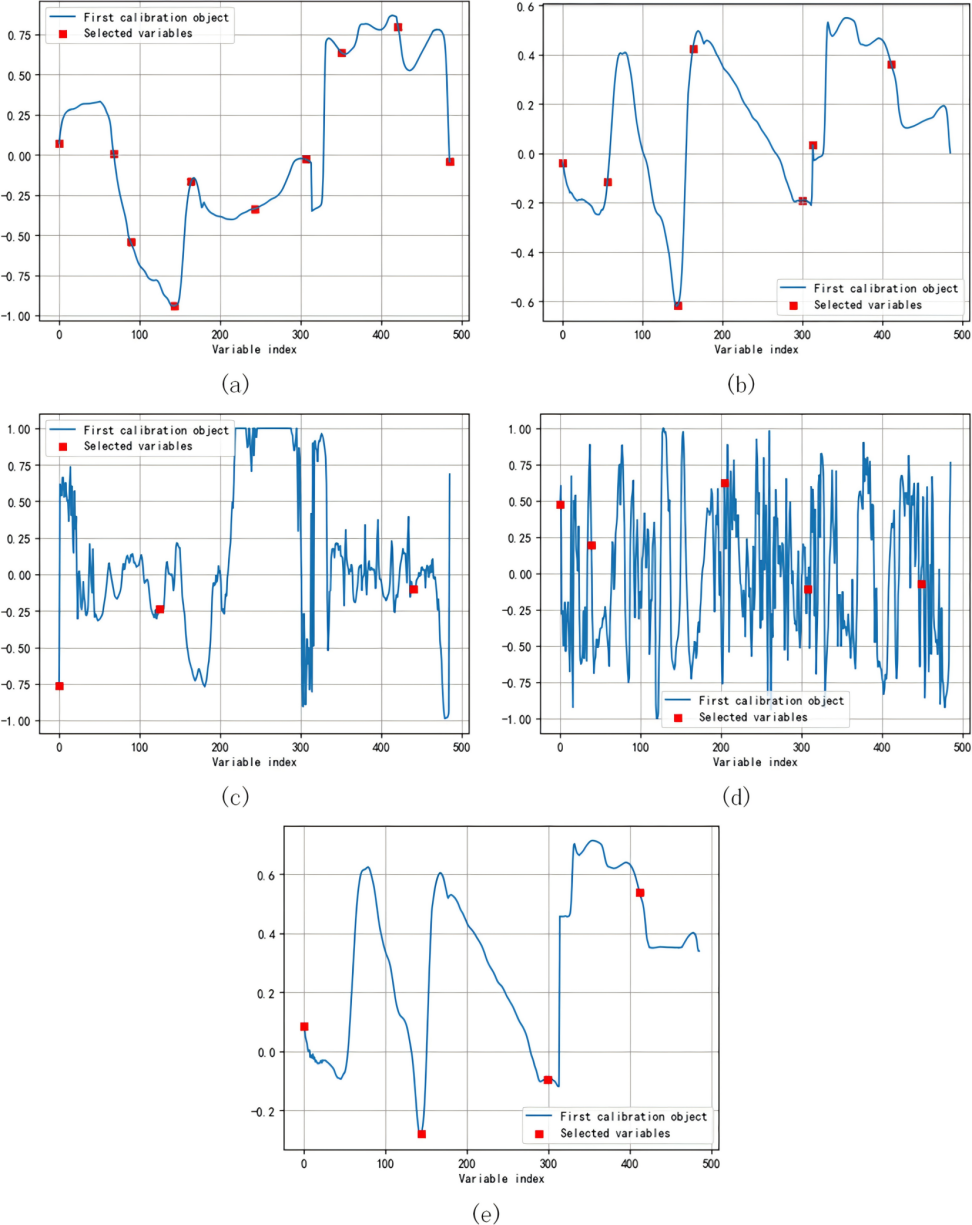
SPA method selects different preprocessed spectral feature wavelength bands. **(a)** Feature selection map after MSC preprocessing; **(b)** Feature selection map after SG preprocessing; **(c)** Feature selection map based on first-order derivative preprocessing; **(d)** Feature selection map based on second-order derivative preprocessing; **(e)** Feature selection map based on logarithmic preprocessing.

In order to facilitate the development of more robust models and their application in multi-spectral imaging systems, band selection is very important. In this study, five pre-treated bands, SG-A, MSC-A, dA, ddA and lgA, were used together to extract the spectral feature bands of pineapple soluble solid by SPA. Due to the randomness of the screening bands by SPA method, intensive screening was performed again by integrating the feature bands selected for five times (that is, to select bands relatively similar as far as possible). Ensure that it does not exceed 10% of the sample size. The selection range of the number of SPA bands is set to 3-10, and the specific number is determined by the number of characteristic bands corresponding to the lowest RMSE value. The number of characteristic wavelengths of MSC-A, SG-A, dA, ddA and lgA screened by SPA were 10, 7, 3, 5 and 4, respectively (**Figure 6**), and the characteristic proportions were 2.057%, 1.440%, 0.061%, 1.028% and 0.823%, respectively. The spectral bands of pineapple fruit were found to be 673-676nm, 711-715nm, 971-990nm and 1357-1367nm. Among them, 673-676nm and 711-715nm are respectively located in the red light and red-edge spectral intervals of the visible light region, while 971-990nm and 1357-1367nm are in the near-infrared region (Cao et al., 2025; Ustin and Jacquemoud, 2020). Existing research has shown that the red-edge is a distinct feature of the plant spectral curve and a key area of focus (Frampton et al., 2013).

### 3.4 Establishment of pineapple SSC prediction model

#### 3.4.1 Construction of SSC prediction model based on full band spectrum

A total of 486 spectral indices of visible light (VL) and near-infrared (NIR) were combined into independent variables, and SSC data was taken as the dependent variable. The random splitting method was used to divide the proportion of training set and test set. Since there were only 100 pineapple samples, the partition ratio was 9:1. A prediction model of SSC metabolites based on PLSR, Lasso and RR was established using the five pre-treated spectra of pineapple fruit as input respectively. During PLSR model building, the parameter “n_components” indicates the number of principal components to be retained, as the independent variable used as a prediction should not exceed 10% of the pineapple sample, set its value to 10, and the remaining parameters are default values. In Lasso model building, “alpha” is the key parameter that controls the regularization strength of the model, punishing the complexity of the model by adding a penalty term to the loss function, thus making the model more simple and robust. Due to the large number of features in this pineapple dataset, set the alpha value to 0.001 and the remaining parameters to their default values. During the construction of the RR model, its “alpha” parameter was set to 0.001 to enhance the generalization ability, and the remaining parameters were default values. The modeling results are shown in **Table 1**. The results showed that SSC had the best effect on PLSR regression prediction under MSC preconditioning, and the test set Equation 1 was 0.9459, Equation 2 was 0.0289, and Equation 3 was 0.5746 (**Table 2**). Compared with the results of existing near-infrared spectroscopy studies (Shiina et al., 1993; Chia et al., 2012; Amuah et al., 2019; Walsh et al., 2020), the MSC-PLSR model adopted here has a better prediction effect.

**Table 2:** SSC prediction model based on different pretreatment of full-band spectrum.

Preconditioning	PLSR			LASSO			RR		
	R^2^	MAPE	RMSE	R^2^	MAPE	RMSE	R^2^	MAPE	RMSE
MSC-A	0.9459	0.0289	0.5746	0.8800	0.2053	1.0175	0.8456	0.1923	1.1144
SG-A	0.8453	0.0636	1.2147	0.8796	0.2534	1.2322	0.8560	0.2438	1.2632
dA	0.8893	0.0495	0.9254	0.8965	0.2148	1.0221	0.8256	0.2815	1.7198
ddA	0.9446	0.0339	0.7136	0.8714	0.1612	0.8185	0.8070	0.2058	1.3174
lgA	0.8445	0.0540	1.0237	0.8650	0.2403	1.2107	0.8814	0.2701	1.2901

#### 3.4.2 Construction of SSC prediction model based on characteristic band spectrum

Five pre-treated bands, SG-A, MSC-A, dA, ddA and lgA, were used together to extract the spectral feature bands of pineapple soluble solid by continuous projection algorithm (SPA). Due to the randomness of the screening bands by SPA method, intensive screening was performed again by integrating the feature bands selected for five times (that is, to select relatively similar bands as far as possible). Ensure that it does not exceed 10% of the sample size. With the characteristic band selected by SPA as independent variable and pineapple SSC as dependent variable, the PLSR, Lasso and RR prediction models of pineapple SSC based on the characteristic band spectrum were established by combining five pretreatment methods. The results show that compared with the full-band model, the prediction accuracy of the feature band is slightly improved, and the prediction results obtained by only the pre-screened feature band dA are inferior to that of the full-band. The reason is that there are only three segments of the screened feature band and too few variables, which leads to the decline of the prediction accuracy. The accuracy of the feature bands decreased slightly under the MSC-PLSR model, while the prediction accuracy reached the highest under the ddA-PLSR model, with R^2^ of 0.9869, RMSE of 0.1250 and MAPE of 0.0058, achieving a relatively good prediction effect (**Table 3**).

**Table 3:** SSC prediction models based on different pretreatments of characteristic band spectra.

Preconditioning	PLSR			LASSO			RR		
	R^2^	MAPE	RMSE	R^2^	MAPE	RMSE	R^2^	MAPE	RMSE
MSC-A	0.9063	0.0218	0.5409	0.9736	0.0914	0.2363	0.9162	0.0534	0.2441
SG-A	0.9269	0.0181	0.3949	0.9255	0.0860	0.3988	0.9265	0.0861	0.3961
dA	0.7522	0.0251	0.4952	0.6910	0.0518	0.4935	0.6936	0.0523	0.4915
ddA	0.9869	0.0058	0.1250	0.8464	0.0543	0.3294	0.8496	0.0547	0.3260
lgA	0.8481	0.0124	0.2464	0.8768	0.0429	0.2207	0.8712	0.0433	0.2256

## 4 Conclusion

This study focused on the demand for non-destructive detection of soluble solids content (SSC) in pineapples, using 100 fruits from 5 pineapple cultivars (‘watermelo’, ‘Josapine’, ‘Tainung No.23’, ‘Tainung No.16’, ‘Tainung No.21’) as research objects. A systematic study on the application of visible-near infrared (400–1700 nm) hyperspectral imaging technology was conducted to provide technical support and theoretical basis for non-destructive quality identification of pineapples.

By optimizing the spectral acquisition and preprocessing process, the study found that multiplicative scatter correction (MSC) had the best effect on eliminating scattering interference in pineapple spectral data, which could effectively highlight the spectral characteristics related to SSC. The full-band partial least squares regression (PLSR) model after MSC preprocessing showed significantly better detection performance (R²=0.9459, RMSE = 0.5746) than Lasso regression (R²=0.8800, RMSE = 1.0175) and ridge regression (RR, R²=0.8456, RMSE = 1.1144), verifying the basic detection capability of hyperspectral technology for pineapple SSC. Furthermore, the Successive Projections Algorithm (SPA) was used to screen characteristic bands from the preprocessed spectra, and four key wavelengths (673–676 nm, 711–715 nm, 971–990 nm, 1357–1367 nm) were successfully extracted: 673–676 nm and 711–715 nm correspond to the absorption characteristics of chlorophyll and carotenoids in pineapple peel/pulp, indirectly reflecting the synergistic changes between fruit maturity and SSC; 971–990 nm and 1357–1367 nm are associated with water molecule vibration and carbohydrate functional group response, respectively, directly pointing to the chemical nature of SSC. This result provides clear wavelength targets for the development of subsequent dedicated multispectral sensors.

The second derivative-PLSR (ddA-PLSR) model constructed based on characteristic bands further eliminated baseline drift and background noise through the second derivative, ultimately achieving a significant improvement in detection accuracy (R²=0.9869, RMSE = 0.1250). At the same time, the stable performance of this model in different pineapple cultivars confirms its certain cultivar universality, providing a feasible solution for cross-cultivar pineapple SSC detection.

In conclusion, this study clarified the technical path of hyperspectral imaging technology for non-destructive detection of pineapple SSC: using MSC as the optimal preprocessing method, and constructing a ddA-PLSR model after screening characteristic bands via SPA, which can balance detection accuracy and practicality. This study not only fills the research gap of “characteristic bands-model optimization-cultivar adaptation” in the field of pineapple hyperspectral quality detection, but also provides a promotable technical paradigm for postharvest quality grading and industrial chain quality traceability of tropical fruits, with important academic value and industrial application prospects.

## Data Availability

The original contributions presented in the study are included in the article/supplementary material. Further inquiries can be directed to the corresponding authors.

## References

[B1] Abraham R. A. Jayasree J. T. Abdullah S. (2023). A comprehensive review of pineapple processing and its by-product valorization in India. Food Chem. Adv. 3, 100416. doi: 10.1016/j.focha.2023.100416

[B2] Aghilinategh N. Dalvand M. J. Anvar A. (2020). Detection of ripeness grades of berries using an electronic nose. Food Sci. Nutre. 8, 4919–4928. doi: 10.1002/fsn3.1788, PMID: 32994953 PMC7500766

[B3] Alcin O. F. Sengur A. Ghofrani S. Ince M. C. (2014). Ga-selm: greedy algorithms for sparse extreme learning machine. Measurement 55, 126–132. doi: 10.1016/j.measurement.2014.04.012

[B4] Ali M. M. Hashim N. Aziz S. A. Lasekan O. O. (2020). Pineapple (ananas comosus): a comprehensive review of nutritional values, volatile compounds, health benefits, and potential food products. Food Res. Int. 137, 109675. doi: 10.1016/j.foodres.2020.109675, PMID: 33233252

[B5] Amuah C. L. Y. Teye E. Lamptey F. P. Nyandey K. Opoku-Ansah J. Adueming O. W. (2019). Feasibility study of the use of handheld nir spectrometer for simultaneous authentication and quantification of quality parameters in intact pineapple fruits. J. Spectrosc., 1–9. doi: 10.1155/2019/5975461

[B6] Ardila C. E. C. Ramirez L. A. Ortiz F. A. P. (2020). Spectral analysis for the early detection of anthracnose in fruits of sugar mango (mangifera indica). Comput. Electron. Agric. 173, 105357. doi: 10.1016/j.compag.2020.105357

[B7] Baiano A. Terracone C. Peri G. Romaniello R. (2012). Application of hyperspectral imaging for prediction of physico-chemical and sensory characteristics of table grapes. Comput. Electron. Agr. 87, 142–151. doi: 10.1016/j.compag.2012.06.002

[B8] Cao C. Deng P. Li J. Lin Y. Fan J. Deng J. . (2025). Construction of hyperspectral regression models for chemical components of tobacco leaves in the field and analysis of spectral characteristics. S-Cent. Agric. Sci. Technol. 46, 38–44.

[B9] Chia K. S. Rahim H. A. Rahim R. A. (2012). Prediction of soluble solids content of pineapple via non-invasive low cost visible and shortwave near infrared spectroscopy and artificial neural network. Biosyst. Eng. 113, 158–165. doi: 10.1016/j.biosystemseng.2012.07.003

[B10] Choo W. S. (2019). Fruit pigment changes during ripening. Encycl. Food Chem., 117–123. doi: 10.1016/B978-0-08-100596-5.21656-9

[B11] Dong J. Guo W. (2015). Nondestructive determination of apple internal qualities using near-infrared hyperspectral reflectance imaging. Food Anal. Methods 8, 2635–2646. doi: 10.1002/jsfa.9360, PMID: 30221355

[B12] Faber Nicolaas M. (1999). Multivariate sensitivity for the interpretation of the effect of spectral pretreatment methods on near-infrared calibration model predictions. Anal. Chem. 71, 557–565. doi: 10.1021/ac980415r, PMID: 21662714

[B13] Fan Y. G. Feng H. K. Liu Y. Bian M. B. Zhao Y. Yang G. J. . (2023). Estimation of nitrogen content in potato plants based on spectral spatial characteristics. Spectrosc. Spectral. Anal. 43, 1532–1540. doi: 10.3964/j.issn.1000-0593(2023)05-1532-09

[B14] FAO (2023). Faostat database. Available online at: https://www.fao.org/faostat/en/data/QCL (Accessed March 18, 2025).

[B15] Frampton W. J. Dash J. Watmough G. Milton E. J. (2013). Evaluating the capabilities of sentinel-2 for quantitative estimation of biophysical variables in vegetation. Isprs. J. Photogramm. Remote Sens. 82, 83–92. doi: 10.1016/j.isprsjprs.2013.04.007

[B16] Goke S. M. S. (2018). Postharvest dry matter and soluble solids content prediction in d’anjou and bartlett pear using near-infrared spectroscopy. HortScience 53, 669–680. doi: 10.21273/hortsci12843-17

[B17] Guo W. Zhao F. Dong J. (2016). Nondestructive measurement of soluble solids content of kiwifruits using near-infrared hyperspectral imaging. Food Anal. Methods 9, 38–47. doi: 10.1007/s12161-015-0165-z

[B18] He M. C. Ma Z. H. (2015). Design of a reference value-based sample-selection method and evaluation of its prediction capability. Chemometr. Intell. Lab. Syst. 148, 72–76. doi: 10.1016/j.chemolab.2015.09.001

[B19] Hoerl A. E. Kennard R. W. (1970). Ridge regression: biased estimation for nonorthogonal problems. Technometrics 12, 55–67. doi: 10.1080/00401706.1970.10488634

[B20] Hossain F. (2016). World pineapple production: an overview. Afr. J. Food Agric. Nutr. Dev. 16, 11443–11456. doi: 10.18697/ajfand.76.15620

[B21] Huang Y. Xiong J. Jiang X. Chen K. Hu D. (2022). Assessment of firmness and soluble solids content of peaches by spatially resolved spec troscopy with a spectral difference technique. Comput. Electron. Agric. 200, 107212. doi: 10.1016/j.compag.2022.107212

[B22] Kabir M. Unal F. Akinci T. C. Martinez-Morales A. A. Ekici S. (2024). Revealing GLCM metric variations across a plant disease dataset: a comprehensive examination and future prospects for enhanced deep learning applications. Electronics 13, 2299. doi: 10.3390/electronics13122299

[B23] Li M. Han D. Liu W. (2019). Non-destructive measurement of soluble solids content of three melon cultivars using portable visible/near infrared spectroscopy. Biosyst. Eng., 188, 31–39. doi: 10.1016/j.biosystemseng.2019.10.003

[B24] Li C. Li M. Zhang M. Chen L. Wu Q. He J. . (2024). Development, prevention, and detection of pineapple translucency: a review. Agronomy. 14, 2755. doi: 10.3390/agronomy14122755

[B25] Li J. Tian X. Huang W. Zhang B. Fan S. (2016). Application of long-wave near infrared hyperspectral imaging for measurement of soluble solid content (ssc) in pear. Food Anal. Methods 9, 3087–3098. doi: 10.1007/s12161-016-0498-2

[B26] Lu Y. Saeys W. Kim M. Peng Y. Lu R. (2020). Hyperspectral imaging technology for quality and safety evaluation of horticultural products: a review and celebration of the past 20-year progress. Postharvest. Biol. Technol. 170, 111318. doi: 10.1016/j.postharvbio.2020.111318

[B27] Ma T. Zhao J. Inagaki T. Su Y. Tsuchikawa S. (2022). Rapid and nondestructive prediction of firmness, soluble solids content, and ph in kiwifruit using vis–nir spatially resolved spectroscopy. Postharvest. Biol. Technol. 186, 111841. doi: 10.1016/j.postharvbio.2022.111841

[B28] Munera S. Amigo J. M. Blasco J. Cubero S. Talens P. Aleixos N. (2017). Ripeness monitoring of two cultivars of nectarine using vis-nir hyperspectral reflectance imaging. J. Food Eng. 214, 29–39. doi: 10.1016/j.jfoodeng.2017.06.031

[B29] Rahim H. A. Seng C. K. Rahim R. A. (2014). Analysis for soluble solid contents in pineapples using NIR spectroscopy. J. Teknol. 69, 7–11. doi: 10.11113/JT.V69.3288

[B30] Reddy R. Pullanagari Li M. (2021). Uncertainty assessment for firmness and total soluble solids of sweet cherries using hyperspectral imaging and multivariate statistics. J. Food Eng. 289, 110177. doi: 10.1016/j.jfoodeng.2020.110177

[B31] Semyalo D. Kwon O. Wakholi C. Min H. J. B. K. C. (2024). Nondestructive online measurement of pineapple maturity and soluble solids content using visible and near-infrared spectral analysis. Postharvest. Biol. Technol. 209, 112706. doi: 10.1016/j.postharvbio.2023.112706

[B32] Shi H. Yu P. (2017). Comparison of grating-based near-infrared (NIR) and Fourier transform mid-infrared (ATR-FT/MIR) spectroscopy based on spectral preprocessing and wavelength selection for the determination of crude protein and moisture content in wheat. Food Ctrl. 82, 57–65. doi: 10.1016/j.foodcont.2017.06.015

[B33] Shiina T. Ijiri T. Matsuda I. Sato T. Kawano S. Ohoshiro N. (1993). Determination of brix value and acidity in pineapple fruits by near infrared spectroscopy. Acta Hortic. 334), 261–272. doi: 10.17660/actahortic.1993.334.27

[B34] Shinzawa H. Ritthiruangdej P. Ozaki Y. (2011). Kernel analysis of partial least squares (pls) regression models. Appl. Spectrosc. 65, 549–556. doi: 10.1366/10-06187, PMID: 21513599

[B35] Tian P. Meng Q. H. Wu Z. F. Lin J. J. Zhu H. Zhou X. L. . (2023). Detection of mango soluble solid content using hyperspectral imaging technology. Infrared. Phys. Technol. 129, 104576. doi: 10.1016/j.infrared.2023.104576

[B36] Ustin S. L. Jacquemoud S. (2020). How the optical properties of leaves modify the absorption and scattering of energy and enhance leaf functionality. doi: 10.1007/978-3-030-33157-3_14

[B37] Walsh K. B. Blasco J. Zude-Sasse M. Sun X. (2020). Visible-nir ‘point’ spectroscopy in postharvest fruit and vegetable assessment: the science behind three decades of commercial use. Postharvest. Biol. Technol. 168, 111246. doi: 10.1016/j.postharvbio.2020.111246

[B38] Wang H. Peng J. Xie C. Bao Y. He Y. (2015). Fruit quality evaluation using spectroscopy technology: A review. Sensors 15, 11889–11927. doi: 10.3390/s150511889, PMID: 26007736 PMC4481958

[B39] Wang B. Yang H. Zhang S. Li L. (2023). Detection of defective features in cerasus humilis fruit based on hyperspectral imaging technology. Appl. Sci. 13, 3279. doi: 10.3390/app13053279

[B40] Wei X. He J. Zheng S. Ye D. (2020). Modeling for ssc and firmness detection of persimmon based on nir hyperspectral imaging by sample partitioning and variables selection. Infrared. Phys. Technol. 105, 103099. doi: 10.1016/j.infrared.2019.103099

[B41] Xie C. Chu B. He Y. (2018). Prediction of banana color and firmness using a novel wavelengths selection method of hyperspectral imaging. Food Chem. 245, 132–140. doi: 10.1016/j.foodchem.2017.10.079, PMID: 29287354

[B42] Xu M. Sun J. Cheng J. Yao K. Wu X. Zhou X. (2023). Non-destructive prediction of total soluble solids and titratable acidity in kyoho grape using hyperspectral imaging and deep learning algorithm. Int. J. Food Sci. Technol. 58, 9–21. doi: 10.1111/ijfs.16173

[B43] Yan M. Wang H. Wu Y. Cao X. Xu H. (2021). Detection of chlorophyll content of Epipremnum aureum based on fusion of spectrum and texture features. J. Nanjing. Agric. Univ. 44, 568–575. doi: 10.7685/jnau.202006013

[B44] Yang X. Chen J. Jia L. Yu W. Wang D. Wei W. . (2020b). Rapid and non-destructive detection of compression damage of yellow peach using an electronic nose and chemometrics. Sensors. 20, 1866. doi: 10.3390/s20071866, PMID: 32230958 PMC7181052

[B45] Yang B. Gao Y. Yan Q. Qi L. Zhu Y. Wang B. (2020a). Estimation method of soluble solid content in peach based on deep features of hyperspectral imagery. Sensors. 20, 5021–5021. doi: 10.3390/s20185021, PMID: 32899646 PMC7570831

[B46] Yu S. Wang N. Ding X. Qi Z. Hu N. Duan S. . (2022). Detection of pear freezing injury by non-destructive x-ray scanning technology. Postharvest. Biol. Technol. 190, 111950. doi: 10.1016/j.postharvbio.2022.111950

[B47] Yuan W. Jiang H. Yang S. Zhang C. Zhou Y. Zhou H. (2024). Geographical origin identification of Lycium barbarum fruit using hyperspectral imaging technology. Food Sci. 45, 254–260. doi: 10.7506/spkx1002-6630-20230620-159

[B48] Zhang X. Sun J. Li P. Zeng F. Wang H. (2021). Hyperspectral detection of salted sea cucumber adulteration using different spectral preprocessing techniques and svm method. LWT- Food Sci. Technol. 152, 112295. doi: 10.1016/j.lwt.2021.112295

[B49] Zhang H. Zhang S. Dong W. Luo W. Huang Y. Zhan B. . (2020). Detection of common defects on mandarins by using visible and near infrared hyperspectral imaging. Infrared Phys. Postharvest. Biol. Technol. 163, 111148. doi: 10.1016/j.postharvbio.2020.111148

